# Genome Mining of α-Pyrone Natural Products from Ascidian-Derived Fungus *Amphichorda* *felina* SYSU-MS7908

**DOI:** 10.3390/md20050294

**Published:** 2022-04-27

**Authors:** Siwen Yuan, Litong Chen, Qilin Wu, Minghua Jiang, Heng Guo, Zhibo Hu, Senhua Chen, Lan Liu, Zhizeng Gao

**Affiliations:** 1 School of Maine Sciences, Sun Yat-sen University, Guangzhou 510006, China; yuansw@mail2.sysu.edu.cn (S.Y.); chenlt28@mail2.sysu.edu.cn (L.C.); wuqlin3@mail2.sysu.edu.cn (Q.W.); jiangmh23@mail2.sysu.edu.cn (M.J.); guoh59@mail2.sysu.edu.cn (H.G.); huzhb6@mail2.sysu.edu.cn (Z.H.); chensenh@mail.sysu.edu.cn (S.C.); cesllan@mail.sysu.edu.cn (L.L.); 2 Southern Laboratory of Ocean Science and Engineering (Guangdong, Zhuhai), Zhuhai 519000, China; 3 Southern Marine Science and Engineering Guangdong Laboratory (Zhuhai), Zhuhai 519000, China; 4 Pearl River Estuary Marine Ecosystem Research Station, Ministry of Education, Zhuhai 519082, China

**Keywords:** genome mining, α-pyrone natural products, heterologous expression, *Aspergillus oryzae* NSAR1, anti-inflammatory activity

## Abstract

Culturing ascidian-derived fungus *Amphichorda felina* SYSU-MS7908 under standard laboratory conditions mainly yielded meroterpenoid, and nonribosomal peptide-type natural products. We sequenced the genome of *Amphichorda felina* SYSU-MS7908 and found 56 biosynthetic gene clusters (BGCs) after bioinformatics analysis, suggesting that the majority of those BGCSs are silent. Here we report our genome mining effort on one cryptic BGC by heterologous expression in *Aspergillus oryzae* NSAR1, and the identification of two new α-pyrone derivatives, amphichopyrone A (**1**) and B (**2**), along with a known compound, udagawanone A (**3**). Anti-inflammatory activities were performed, and amphichopyrone A (**1**) and B (**2**) displayed potent anti-inflammatory activity by inhibiting nitric oxide (NO) production in RAW264.7 cells with IC_50_ values 18.09 ± 4.83 and 7.18 ± 0.93 μM, respectively.

## 1. Introduction

Fungal natural products are an indispensable source for drug development [1,2]. However, under typical laboratory culture conditions, most of the biosynthetic gene clusters (BGCs) from fungi are “silent”; thus, it becomes increasingly difficult to discover novel natural products. Currently, several genome mining strategies have been reported for activating silent BGCs [3], and heterologous expression of target BGCs in a suitable host is one effective approach. Zhu et al. [4] expressed a cryptic BGC from *Trichoderma harzianum* t-22 in *Aspergillus nidulans* A1145 and successfully isolated several tetronate natural products, such as trihazone A–F. Similarly, Li et al. [5] characterized a cryptic BGC from *A. hancockii* in *A. nidulans* LO8030 and discovered a metabolite with a unique prenylated 6/6/6/5 tetracarbocyclic skeleton. The quadruple auxotroph *A. oryzae* NSAR1 is also a frequently-used heterologous host for genome mining of fungal natural products. Recently, Jiang et al. [6] used *A. oryzae* as a heterologous host to express two fungal bifunctional terpene synthases and obtained four terpenes featuring 5-6-7-3-5 ring systems; Yan et al. [7] genome mined four new meroterpenoids, funiculolides A−D, by heterologous expression of a cryptic BGC from *A. funiculosus* CBS 116.56.

Discovering novel secondary metabolites from marine-derived fungi has been our group’s long-term research interest [8,9,10,11]. The fungus *Amphichorda felina* SYSU-MS7908 was isolated from a marine ascidian *Styelaplicata* and fermentation under standard laboratory conditions mainly gave meroterpenoids and nonribosomal peptides [12,13]. To probe the biosynthetic potential of *A. felina* SYSU-MS7908, the genome was sequenced by Illumina second-generation sequencing, and the assembled genome was analyzed by the antibiotics and secondary metabolite analysis shell (antiSMASH) [14]. Fifty-six secondary metabolites BGCs were found (Figure S1), suggesting that the fungus has great potential to produce structurally diverse natural products.

**Figure 1:**
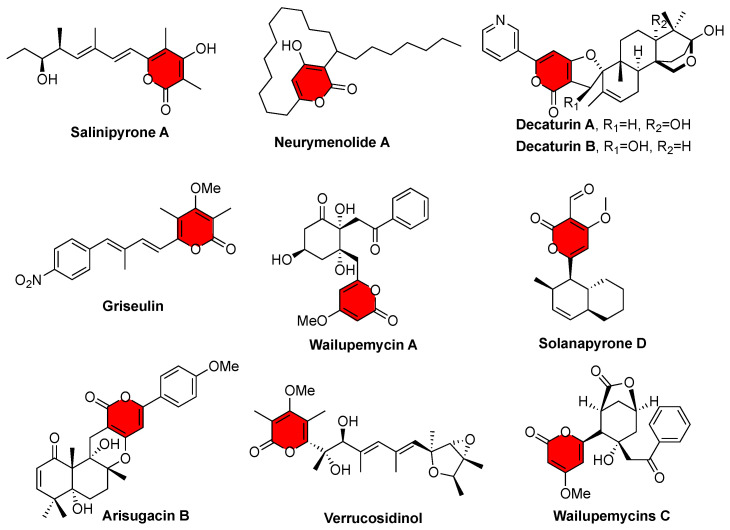
Representative natural products containing α-pyrone motif.

**Figure 2:**
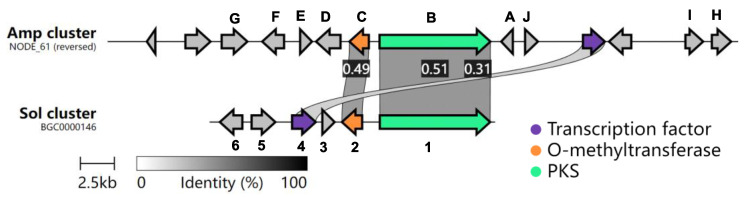
Comparison of the *amp* cluster with the *sol* cluster was performed using the Clinker tool [22].

Pyrones are six-membered cyclic unsaturated esters exhibiting a broad range of activities, such as antifungal, antibiotic, cytotoxic, immunosuppressive, and phytotoxic (Figure 1) [15,16,17]. One gene cluster, the *amp* cluster discovered from *A. felina* SYSU-MS7908, shares similarities with the *sol* cluster that is responsible for the biosynthesis of solanapyrone D (Figure 2), an α-pyrone containing natural product [18]. However, we had not observed the production of such metabolites from the wild type of *A. felina* SYSU-MS7908, suggesting that this BGC might be silent. We decided to activate this BGC by heterologous expression in *A. oryzae* NSAR1 as it is a robust host to mine fungal natural products [19,20,21].

## 2. Results and Discussion

### 2.1. Bioinformatic Analysis of the Amp Cluster

The *amp* cluster contains 10 potential biosynthetic genes (transcription factor and transport excluded, Table 1). AmpB is a polyketide synthase (PKS) that shares 52% protein identity with Sol1 (Figure S2), the PKS involved in solanapyrone D biosynthesis [18,23,24]. AmpC, a putative methyltransferase, shares 49.5% protein identity with Sol2 (Figure S3). Interestingly, no other genes share significant sequence homology between those two clusters (Figure 2), suggesting that the *amp* cluster might produce structurally divergent natural products from solanapyrone D.

### 2.2. Heterologous Expression of the Amp Cluster in A. oryzae

**Figure 3:**
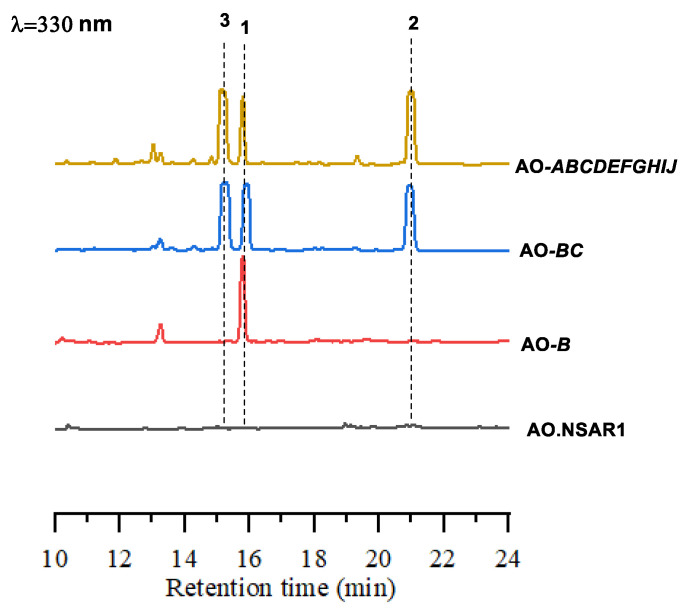
HPLC profiles obtained after expression of respective constructs in *A. oryzae*.

Previous studies suggested that the PKS, Sol1, could produce an advanced biosynthetic intermediate; thus, only the *ampB* gene was introduced into the *A. oryzae* NSAR1 host strain. As expected, the production of amphichopyrone A (**1**) was detected from the expression of the AO-*ampB* construct (Figure 3). With amphichopyrone A (**1**) determined, we next focused on the tailoring genes of the *amp* cluster. AmpC, a putative *O*-methyltransferase, was then introduced into AO-*ampB* to give the construct AO-*ampBC*. We found that two additional metabolites were produced by AO-*amp*BC. Large-scale fermentation and spectroscopic analyses determined the structures as amphichopyrone B (**2**) and udagawanone A (**3**) [15,16,17].

**Figure 4:**
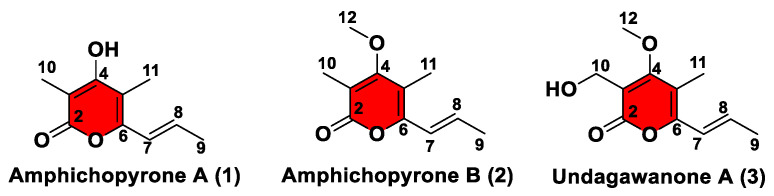
Chemical structures of **1**–**3**.

**Figure 5:**
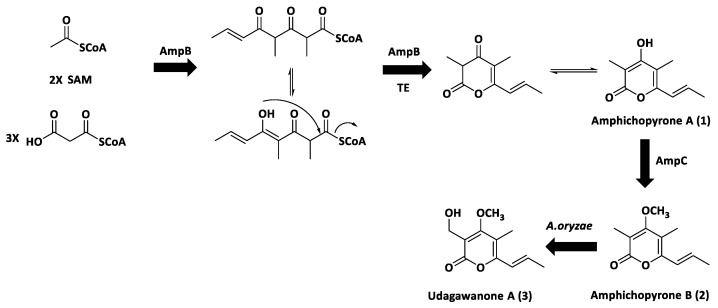
The biosynthetic pathway of **1**–**3**. AmpB is responsible for the formation of amphichopyrone A (**1**); AmpC introduces a methyl group to give amphichopyrone B (**2**) by AmpC; hydroxylation of amphichopyrone B (**2**) to udagawanone A (**3**) might be catalysed by endogenous enzymes from *A. oryzae* NSAR1 host.

To investigate the final metabolites produced by the *amp* cluster, the remaining eight genes, *ampADEFGHIJ,* were included in the construct; unfortunately, the AO-*ampABCDEFGHIJ* construct generated the same product profile as AO-*ampBC* (Figure 3), suggesting amphichopyrone (**2**) and udagawanone A (**3**) might be the final products catalyzed by the *amp* cluster (Figure 4). These results suggest that AmpC catalyzed the methylation reaction at C-4 hydroxyl of amphichopyrone A (**1**) to give amphichopyrone B (**2**). Hydroxylation of amphichopyrone B (**2**) to udagawanone A (**3**) might be catalyzed by endogenous enzymes from *A. oryzae* NSAR1 host (Figure 5).

### 2.3. Characterization of amphichopyrone A (1) and B (2)

**Figure 6:**
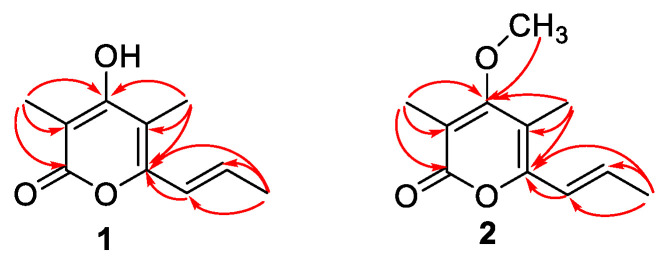
The key HMBC (red arrow) correlations of compounds **1** and **2**.

Amphichopyrone A (**1**) was obtained as a white solid. The positive HR-ESI-MS gave an *m/z* 181.0860 [M+H]^+^, consistent with the molecular formula C_10_H_13_O_3_ with five degrees of unsaturation (Figure S4). The IR absorption spectrum suggested the presence of hydroxyl (3545 cm^−1^), conjugated carboxyl ester (1668 cm^−1^), and olefinic (1568 cm^−1^) functional groups in the molecule (Figure S5). The UV maxima at approximately 222 and 325 nm suggested the presence of an α-pyrone substructure (Figure S6) [17,25,26]. The ^1^H NMR, DEPT NMR, and HSQC experiments showed two olefinic proton signals [δ_H_ 6.42 (1H, dq, J = 15.4, 1.3 Hz)/δ_C_ 120.6 and 6.50 (1H, dq, J = 15.4, 6.0 Hz)/δ_C_ 132.0] and three methyls [δ_H_ 1.90 (3H, d, J = 6.0 Hz)/δ_C_ 17.6; δ_H_ 1.94 (3H, s)/δ_C_ 8.6 and δ_H_ 2.01 (3H, s)/δ_C_ 8.5] (Table 2). Except for the above-mentioned five carbons, the ^13^C NMR spectrum and HSQC experiment revealed the presence of five non-protonated carbons, which were attributed to the 3,4,5,6-tetrasubstituted α-pyrone skeleton (δ_C_ 99.0, 106.3, 151.6, 163.6, 164.3), consisting of one carbonyl carbon and four sp^2^ carbons. The HMBC correlations of CH_3_-10 to C-2 (*δ*_C_ 163.6), C-3 (*δ*_C_ 99.0) and C-4 (*δ*_C_ 164.3), CH_3_-11 to C-4 (*δ*_C_ 164.3), C-5 (*δ*_C_ 106.3) and C-6 (*δ*_C_ 151.6), and CH_3_-9 to C-6 (*δ*_C_ 151.6), C-7 (*δ*_C_ 120.6) and C-8 (*δ*_C_ 132.0), which suggested the presence of methyl at C-3 and C-5 site in α-pyrone, and the connection of C-6 site in α-pyrone to the propenyl chain (Figure 6). The coupling constant (15.4 Hz) of two olefinic protons established the *trans* geometric configuration for ∆^7(8)^. Hence, the structure of compound **1** was elucidated as (*E*)-4-hydroxy-3,5-dimethyl-6-(prop-1-en-1-yl)-2*H*-pyran-2-one and named amphichopyrone A (**1**).

Amphichopyrone B (**2**) was obtained as a white solid. The molecular formula was determined as C_11_H_15_O_3_ based on the positive HR-ESIMS ions at *m*/*z* 195.1019 [M+H]^+^ (calcd. for 195.1016, C_11_H_15_O_3_), suggesting five degrees of unsaturation (Figure S7). The IR absorption bands at 3394, 1668, and 1568 cm^−1^, indicated the presence of hydroxyl, conjugated carboxyl ester, and olefinic groups in the molecule (Figure S8). Its UV spectrum (λ_max_ 227 and 332 nm) was indicative of the presence of α-pyrone substructure (Figure S9) [18,25,26]. The NMR and HSQC experiments showed two olefinic proton signals [δ_H_ 6.42 (1H, dq, J = 15.4 1.3 Hz)/δ_C_ 121.4 and 6.51 (1H, dq, J = 15.4, 6.5 Hz)/δ_C_ 133.1] and four methyls [δ_H_ 1.91 (3H, d, J = 6.5 Hz)/δ_C_ 18.6; δ_H_ 1.96 (3H, s)/δ_C_ 10.4; δ_H_ 1.98 (3H, s)/δ_C_ 9.5 and δ_H_ 3.83 (3H, s)/δ_C_ 60.7] (Table 2). Except for the above-mentioned six carbons, the ^13^C NMR spectrum and HSQC experiment revealed the presence of five non-protonated carbons, which were attributed to the 3,4,5,6-tetrasubstituted α-pyrone skeleton (δ_C_ 109.4, 111.2, 153.0, 164.5, 168.7), consisting of one carbonyl carbon and four sp^2^ carbons. The HMBC correlations of CH_3_-10 to C-2 (*δ*_C_ 164.5), C-3 (*δ*_C_ 111.2) and C-4 (*δ*_C_ 168.7), CH_3_-11 to C-4 (*δ*_C_ 168.7), C-5 (*δ*_C_ 109.4) and C-6 (*δ*_C_ 153.0), OCH_3_-12 to C-4 (*δ*_C_ 168.7) and CH_3_-9 to C-6 (*δ*_C_ 153.0), C-7 (*δ*_C_ 121.4) and C-8 (*δ*_C_ 133.1), which suggested the presence of methyl at C-3 and C-5 site, methoxy at C-4 site in α-pyrone, and the connection of C-6 site in α-pyrone to the propenyl chain (Figure 6). The coupling constant (15.4 Hz) of two olefinic protons established the *trans* geometric configuration for ∆^7(8)^. Hence, the structure of compound **2** was elucidated as (*E*)-4-methoxy-3,5-dimethyl-6-(prop-1-en-1-yl)-2H-pyran-2-one and named amphichopyrone B (**2**).

### 2.4. Evaluation of Anti-Inflammatory Activity

**Figure 7:**
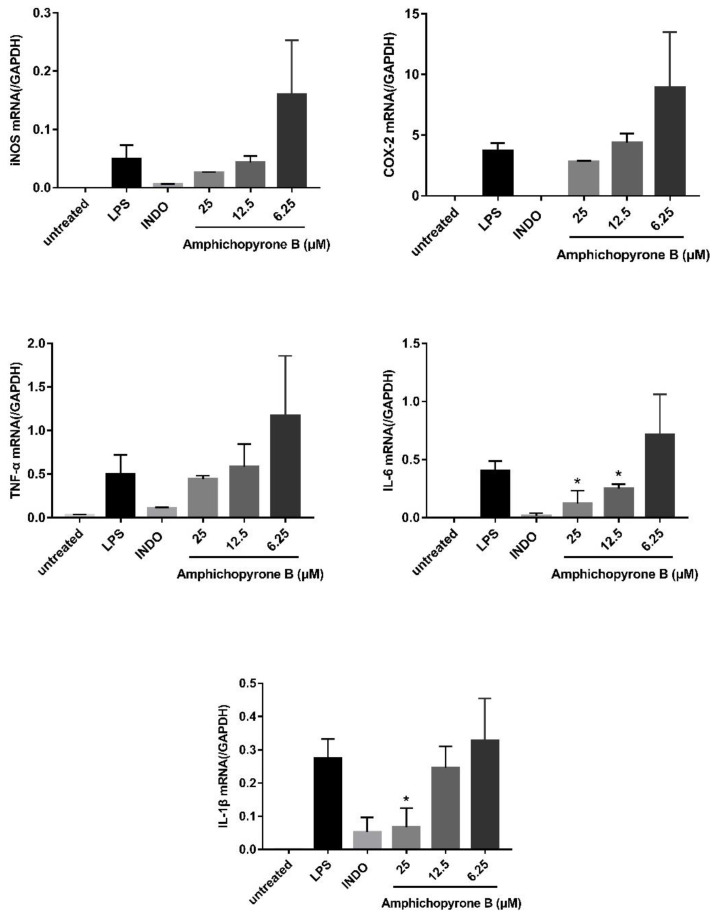
The effect of amphichopyrone B (**2**) on the LPS-induced iNOS, COX-2, TNF-α, IL-6, and IL-1β expression. The ‘untreated’ bar is cells only treated with 0.2% DMSO, the ‘LPS’ bar is cells treated with LPS (1 µg/mL) with 0.2% DMSO, the ‘INDO’ bar is cells treated with indomethacin (50 µM) together and LPS (1 µg/mL), and 25 µM\12.5 µM\6.25 µM represents cells treated with three concentrations of amphichopyrone B (**2**) together with LPS (1 µg/mL). The mRNA expression levels of iNOS, COX-2, TNF-α, IL-6, and IL-1β were down-regulated, suggesting that the LPS-induced inflammatory processes were inhibited by amphichopyrone B. * *p* < 0.05 vs. LPS treatment group.

Compounds **1**–**3** were evaluated for in vitro anti-inflammatory activity. Amphichopyrone A (**1**) and B (**2**) displayed potent anti-inflammatory activity by inhibiting LPS-induced NO production with IC_50_ values 18.09 ± 4.83 and 7.18 ± 0.93 μM, respectively. Interestingly, udagawanone A (**3**) shows no activity up to 50 μM, suggesting the C-10 hydroxyl group is detrimental for its anti-inflammatory activity. The pro-inflammatory enzymes, inducible nitric oxide synthase (iNOS) and cyclooxygenase-2 (COX-2), and transcriptional regulators, tumor necrosis factor-α (TNF-α), interleukin-6 (IL-6), and interleukin-1β (IL-1β), have been shown to play key roles in inflammatory processes [27,28]. Thus, qPCR experiments were conducted to detect the effect of amphichopyrone B on the activated iNOS, COX-2, TNF-α, IL-6, and IL-1β. The results showed that the mRNA expression levels of iNOS, COX-2, TNF-α, IL-6, and IL-1β were down-regulated with the increased concentration of amphichopyrone B (Figure 7), indicating that LPS-induced inflammatory process was inhibited by amphichopyrone B.

## 3. Materials and Methods

### 3.1. General Materials

Chemicals were purchased from Sangon Biotech Co., Ltd. (Shanghai, China), Thermo Fisher Scientific (Shanghai, China), Sigma-Aldrich Trading Co., Ltd. (Shanghai, China) or J&K Scientific Ltd (Beijing, China). unless noted otherwise. Column Chromatography (CC) was carried out using silica gel (200–300 mesh, Qingdao Marine Chemical Factory, Qingdao, China).

DNA Sequence analysis and primer synthesis were performed by TsingKe Biological Technology Co., Ltd. (Guangzhou, China). Plasmid extraction kits and DNA purification kits were purchased from Tianjin Biotech Co., Ltd. (Beijing, China). PCR analysis was accomplished using a Bio-Rad CFX™ Thermal Cycle with Phanta Super-Fidelity DNA Polymerase (Vazyme, Nanjing, China). The assembly of DNA fragments and the construction of recombinant plasmids were performed by using Clone Express^®^ MultiS One Step Cloning Kit (Vazyme, Nanjing, China). Yatalase™ was purchased from Takara Co., Ltd. (Dalian, China). UV spectra were collected on Waters 2998 photodiode array detector (Waters, Boston, MA, USA). NMR spectra were obtained on a Bruker Avance 400 MHz (Bruker, Switzerland) with tetramethylsilane (TMS) as the internal standard. HR-ESIMS data were measured on a Thermos LCQ DECA XP plus mass spectrometer using a Luna 5u C18 (2) 100A 150×4.60 mm 5-micron column (Thermos Scientific, Waltham, MA, USA). The Semi-preparative and analytical HPLC were performed on a Waters 1525 system equipped with a Waters 2998 photodiode array detector (Waters, Boston, MA, USA), using a Welch Ultimate^®^ XB-C18 column (10 mm × 250 mm, 5 μm, Welch Materials, Inc., Shanghai, China) and COSMOSIL 5C18-AR-II column (4.6 mm × 250 mm, 5 μm, NacalaiTesque, Inc., Kyoto, Japan), respectively. The mobile phases for analytical HPLC were H_2_O containing 0.1% formic acid (A) and CH_3_CN containing 0.1% formic acid (B), and the gradient elution was 15–80% B (0–15 min), 80–100% B (15–20 min), 100–100% B (20–28 min), 100–15% B (28–30 min), and 15% B (30–33 min) with a flow rate of 1 mL/min.

### 3.2. Strains and Media

The strain, *Amphichorda felina* SYSU-MS7908, was isolated from a marine ascidian *Styelaplicata* collected from the north atoll of the Xisha Islands, South China Sea, China, in 2018, isolated using the standard protocol [29] and identified by the morphological and the internal transcribed spacer (ITS) of the nuclear ribosomal DNA data analysis (Accession number MT786206). The whole genome sequences of *A. felina* SYSU-MS7908 have been deposited in the GenBank database with an accession number JAEMHR000000000. The sequences of *amp A-J* have been submitted to GenBank (OL906410-OL906419). The proposed functions of ampA-J in Table 1 are based on the protein BLAST results. The gene cluster comparison figure between the *amp* and *sol* clusters was generated with the Clinker tools with the corresponding annotated sequence files using default parameters [22]. Primer sequences are listed in Table S1.

The heterologous expression host *Aspergillus oryzae* NSAR1 and corresponding plasmids were gifts from professors Ikuro Abe and Katsuhiko Kitamoto (the University of Tokyo, Tokyo, Japan) [30]. The mycelia of *A. oryzae* strains expressing the respective constructs were inoculated into 10 mL DPY medium (2% dextrin, 1% polypeptide, 0.5% yeast extract, 0.5% KH_2_PO_4_, 0.05% MgSO_4_.7H_2_O) and cultured at 28 °C and 220 rpm for 2 days. Then the DPY medium was transferred to the 100 mL modified Czapek-Dox (CD) medium (0.3% NaNO_3_, 0.2% KCl, 0.05% MgSO_4_.7H_2_O, 0.1% KH_2_PO_4_, 0.002% FeSO_4_.7H_2_O, 1% polypeptide, 2% starch, pH 5.5), and grown at 28 °C and 220 rpm for 6 days to induce the expression of heterologous genes under the *amyB* promoter.

### 3.3. Construction of Recombinant Plasmids

To construct the expression plasmids for *A. oryzae*, the genes (*ampA*-*ampJ*) of the *amp* cluster were first amplified using the genomic DNA of *Amphichorda felina* SYSU-MS7908 as a template. Each amplified DNA fragment was then introduced into the pTAex3 vector [31], and the gene expression cassette, *amyB* promoter, the target gene, and the *amyB* terminator were amplified from the pTAex3-based plasmid. These gene expression cassettes were then inserted into the HindIII-linearlized pPTRI [32], pBARI [33], SpeI-linearized pAdeA [34], and XbaI-linearized pUNA [35]. Plasmids constructed in this study are listed in Table S2.

### 3.4. Transformation of A. oryzae NSAR1

The transformation of *A. oryzae* NSAR1 was conducted via the protoplast−polyethylene glycol method. Mycelia of the parent strain from the solid culture in potato dextrose agar were inoculated in 10 mL DPY medium and cultured at 28 °C and 220 rpm for 2 days, which were then transferred to 100 mL DPY medium and cultured for 24 h. The mycelia were collected by filtration and digested by 1% Yatalase in 0.6 M (NH_4_)_2_SO_4_, 50mM maleic acid, pH 5.5 at 30 °C for 4 h to remove cell walls. The resulting protoplasts were collected by centrifugation at 1500 rpm for 10 min and washed once with Solution 2 (1.2 M sorbitol, 50 mM CaCl_2_.2H_2_O, 35 mM NaCl, 10M Tris-HCl, pH 7.5). Then, 200 μL protoplast suspension (1 × 10^7^ cells/mL) and about 10 μg plasmids were gently mixed and incubated on ice for 30 min, followed by the addition of 1.3 mL Solution 3 (60% PEG4000, 50 mM CaCl_2_.2H_2_O, 10 M Tris-HCl, pH 7.5) at three times. After the mixture was placed at room temperature for 20 min, 5 mL Solution 2 was added. Following centrifugation at 1500 rpm for 10 min, the precipitates were suspended in 200 μL of Solution 2 and spread on the bottom selective medium with 0.8% agar, which was then covered with the selective overlay medium containing 1.5% agar. The selective medium was composed of 0.2% NH_4_Cl, 0.1% (NH_4_)_2_SO_4_, 0.05% KCl, 0.05% NaCl, 0.1% KH_2_PO_4_, 0.05% MgSO_4_.7H_2_O, 0.002% FeSO_4_.7H_2_O, 2% glucose, 1.2 M sorbitol supplemented with 0.15% methionine, 0.1% arginine, 0.01% adenine, 0.1 μg/mL pyrithiamine hydrobromide and 35 μL/mL glufosinate-ammonium based on the plasmids used. The *A. oryzae* strain containing the *amp* cluster was verified by checking the relevant exogenous target genes using PCR analysis (Figure S10). *A. oryzae* transformants constructed in this study are listed in Table S3.

### 3.5. Extraction, Isolation, and Characterization

The strain expressing the AO-*ampBC* construct was cultured in a CD-starch medium (3.0 L) at 28 °C and 220 rpm for 5 days. The culture was extracted with ethyl acetate (3 × 3.0 L). The crude extract was subjected to silica column chromatography eluted with CH_2_Cl_2_-methanol (*v*/*v*, 100:10). The fraction was purified by RP-HPLC using 55% methanol in water, a flow rate of 3 mL/min, a Welch Ultimate^®^ XB-C18 column (10 mm × 250 mm, 5 μm, Welch) to afford three white solids **1** (10 mg), **2** (15 mg), and **3** (14 mg).

### 3.6. Anti-Inflammatory Activity

The RAW264.7 cells were used to evaluate the anti-inflammatory activity of compounds **1**–**3** following a literature procedure [36]. The cells were seeded in 96-well plates at a density of 5 × 10^5^ cells/mL. After 12 h, LPS (1 µg/mL) and samples were added to the cells and then incubated for 24 h at 37 °C. The quantity of nitrite accumulated in the culture medium was measured as an indicator of NO production. Then, 50 µL of cell culture medium with 100 µL Griess reagent were mixed and incubated for 10 min at room temperature. The absorbance was determined at 540 nm wavelength with a microplate reader.

### 3.7. Quantification of the Expression of iNOS, COX-2, TNF-α, IL-6, and IL-1β and GAPDH

The RAW264.7 cells were precultured in 6-well plates at a density of 1.0 × 10^6^ cells/mL for 12 h. The cells were then treated with LPS (1 µg/mL), indomethacin (50 µM) together with LPS (1 µg/mL), three concentrations (6.25, 12.5, 25 µM) of amphichopyrone B (**2**) together with LPS (1 µg/mL) for 24 h at 37 °C under a 5% CO_2_ atmosphere. The negative control (untread group) was treated only with 0.2% DMSO. The total RNA was isolated using the RNeasy kit (TransGen Biotech Co., Ltd., Beijing, China). The FastKing gDNA Dispelling RT SuperMix (TransGen Biotech Co., Ltd., Beijing, China) was used for reverse transcription at 42 °C for 15 min and 95 °C for 3 min. The BIO-RAD CFX96 Real-Time system (Bio-Rad Laboratories, Inc., Hong Kong, China) and SYBR Green Premix Pro Taq HS qPCR kit (Accurate Biology Co., Ltd., Changsha, China) were used for qPCR amplification of iNOS, COX-2, TNF-α, IL-6, and IL-1β and GAPDH (40 cycles at 95 °C for 5 s and 60 °C for 30 s).

## 4. Conclusions

Many genome mining strategies have been reported for activating silent BGCs to produce novel natural products. In this study, we heterologously expressed a cryptic gene cluster from *Amphichorda felina* SYSU-MS7908 in *A. oryzae* NSAR1 and two new metabolites, amphichopyrone A (**1**) and amphichopyrone B (**2**), along with a known compound, udagawanone A (**3**), were isolated. Amphichopyrone A (**1**) and amphichopyrone B (**2**) show potent anti-inflammatory activity by inhibiting LPS-induced NO production with IC_50_ values 18.09 ± 4.83 and 7.18 ± 0.93 μM, respectively.

## Figures and Tables

**Table 1:** Proposed functions of open reading frames in the *amp* cluster.

Amp	AA	Homolog (Accession No.)	S/I ^a^ (%)	Proposed Function
A	285	-	-	Methyltransferase
B	2633	Sol1 (D7UQ44.1)	67.5/51.0	polyketide synthase
C	455	Sol2 (XP_045265976.1)	64.6/49.5	*O*-methyltransferase
D	583	1A4 (CRG92717.1)	85.6/72.9	p450
E	273	DltE (XP_045265975.1)	86.4/72.5	Oxidoreductase
F	518	ChyH (XP_037172406.1)	80.6/67.4	FAD-linked oxidoreductase
G	487	—	—	p450
H	422	—	—	Unknown
I	398	—	—	Unknown
J	253	CsgA (CZT51343.1)	70.8/52.2	short-chain dehydrogenase/reductase

^a^ Similarity/identity, AA: Amino acid.

**Table 2:** ^1^H (400 MHz) and ^13^C (100 MHz) NMR data of **1** and **2** in acetone-*d_6_*.

No	1		2	
	*δ*_H_,(*J* in Hz)	*δ*_C,_ Type	*δ*_H_,(*J* in Hz)	*δ*_C,_ Type
2		163.6, C		164.5, C
3		99.0, C		111.2, C
4		164.3, C		168.7, C
5		106.3, C		109.4, C
6		151.6, C		153.0, C
7	6.42, dq (15.4, 1.3)	120.6, CH	6.42, dq (15.4, 1.3)	121.4, CH
8	6.50, dq (15.4, 6.0)	132.0, CH	6.51, dq (15.4, 6.5)	133.1, CH
9	1.90, d (6.0)	17.6, CH_3_	1.91, d (6.5)	18.6, CH_3_
10	1.94, s	8.6, CH_3_	1.96, s	10.4, CH_3_
11	2.01, s	8.4, CH_3_	1.98, s	9.5, CH_3_
12			3.83, s	60.7, CH_3_

## Data Availability

Not applicable.

## Supplementary Materials

The following supporting information can be downloaded at: https://www.mdpi.com/article/10.3390/md20050294/s1, Table S1: Primers used in this study; Table S2: Plasmids constructed in this study; Table S3: *A. oryzae* transformants constructed in this study; Table S4: ^1^H NMR and ^13^C NMR data of; Figure S1: The antiSMASH (fungal version) result of *Amphichorda felina* SYSU-MS7908; Figure S2: The UV spectrum of Amphichopyrone A (1); Figure S3: The UV spectrum of Amphichopyrone B (2); Figure S4: The UV spectrum of undagawanone A (3); Figure S5: The IR spectrum of Amphichopyrone A (1); Figure S6: The IR spectrum of Amphichopyrone B (2); Figure S7: HRESIMS spectrum for Amphichopyrone A (1); Figure S8: HRESIMS spectrum for Amphichopyrone B (2); Figure S9: PCR analysis for the AO-*ampABCDEFGHIJ* construct.; Figure S10: Amino acid sequence alignment of AmpB with Sol1; NMR Spectra.
