# Supplementary material for: Genome Mining of α-Pyrone Natural Products from Ascidian-Derived Fungus Amphichorda felina SYSU-MS7908
Source: Mar Drugs. 2022 Apr 27;20(5):294. doi: 10.3390/md20050294 (PMC9146101; doi:10.3390/md20050294)
Supplement: Supplementary file 1 [file marinedrugs-20-00294-s001.zip › marinedrugs-1666687-supplementary.pdf]

Genome Mining of α-pyrone Natural Products from the Ascidian-Derived Fungus *Amphichorda felina* SYSU-MS7908

Siwen Yuan [1,†], Litong Chen [1,†], Qilin Wu [1], Minghua Jiang [1], HengGuo [1] , Zhibo Hu [1], Senhua Chen [1,2], Lan Liu [1,2,3,4] and Zhizeng Gao [1,2,3,4]

1 School of Maine Sciences, Sun Yat-sen University, Guangzhou 510006, China;

2 Southern Laboratory of Ocean Science and Engineering (Guangdong, Zhuhai), Zhuhai 519000, China

3 Southern Marine Science and Engineering Guangdong Laboratory (Zhuhai), Zhuhai 519000, China

4 Pearl River Estuary Marine Ecosystem Research Station, Ministry of Education, Zhuhai 519082, China

※ Correspondence: gaozhizeng@mail.sysu.edu.cn

† These authors contributed equally to this work.

Contents

Table S1 Primers used in this study 3
Table S2 Plasmids constructed in this study 4
Table S3 *A. oryzae* transformants constructed in this study 5
Table S4 ^{1}H NMR and ^{13}C NMR data of udagawanone A (**3**) 5
Figure S1 The antiSMASH (fungal version) result of *Amphichordafelina* SYSU-MS7908 5
Figure S2 Amino acid sequence alignment of AmpB with Sol1 6
Figure S3 Amino acid sequence alignment of AmpC with Sol2 6
Figure S4 HRESIMS spectrum of Amphichopyrone A (**1**) 7
Figure S5 The IR spectrum of Amphichopyrone A (**1**) 7
Figure S6 The UV spectrum of Amphichopyrone A (**1**) 8
Figure S7 HRESIMS spectrum for Amphichopyrone B (**2**) 8
Figure S8 The IR spectrum of Amphichopyrone B (**2**) 9
Figure S9 The UV spectrum of Amphichopyrone B (**2**) 9
Figure S10 PCR analysis for the AO-*ampABCDEFGHIJ* construct 9
NMR spectra 10

Table S1. Primers used in this study

Primer	Sequence (5' to 3')
pUSA_*A*-F	CTCCGAATTCGAGCTCGGTACCCTGAGTAAAGCATCTCCAGCTC
pUSA_*A*-R	CTACTACAGATCCCCGGGTACCCGGTATATTTTCTCTCTGGCATGC
pTAex3_*B*-F	AGCTCCGAATTCGAGCTCGGTACCATCGTCAGTCGTCAAAGCGAAG
pTAex3_*B*-R	CACGAGCTACTACAGATCCCCGGGCAATCATCGAAAGCTTTTTGGCTCA
pUSA_*C*-F	CAAGCTGAATTCGAGCTCGGTACCTACTGCCTCAAGATTCGCTGGT
pUSA_*C*-R	CACGAGCTACTACAGATCCCCGGGCTGCGAGCCGATGTGACCCC
pUSA_*D*-F	CTCCGAATTCGAGCTCGGTACCACTCGCCAATCTGCCAAATATTCAT
pUSA_*D*-R	CACGAGCTACTACAGATCCCCGGGCTCACAAAGCACTGCTGCG
pTAex3_*E*-F	AGCTCCGAATTCGAGCTCGGTACCGCTGACCATTGCTTCAACACC
pTAex3_*E*-R	CACGAGCTACTACAGATCCCCGGGATGCGAGACTCCCAAATAGTGAG
pTAex3_*F*-F	AGCTCCGAATTCGAGCTCGGTACCGCAGCTAAGCCGTGTCCAG
pTAex3_*F*-R	CACGAGCTACTACAGATCCCCGGGTACAGGTTCTCAAACAGCCAAGGCA
pTAex3_*G*-F	AGCTCCGAATTCGAGCTCGGTACCACGATGTACTAAGATCCGGCCG
pTAex3_*G*-R	CACGAGCTACTACAGATCCCCGGGCATACGTGATATCTGTCTCGGTGG
pTAex3_*H*-F	CTCCGAATTCGAGCTCGGTACCTTCAGCCTTATCCCTGGGTGC
pTAex3_*H*-R	CTACTACAGATCCCCGGGTACCATGAATGTGGGGATTCAGGTT
pTAex3_*I*-F	CTCCGAATTCGAGCTCGGTACCATTACAAGCAAAACAGGCAACCT
pTAex3_*I*-R	CTACTACAGATCCCCGGGTACCGCACCCAGGGATAAGGCTGA
pTAex3_*J*-F	CAAGCTGAATTCGAGCTCGGTACCGCTGAGCAGGCTGAGCAGGG
pTAex3_*J*-R	CACGAGCTACTACAGATCCCCGGGCGACTCTAGGAGTTCCTGTTTAGGT
Infu_pAdeA_SpeI_F	TAGAGGATCTACTAGTCAAGAGCAGAATGTGAACG
Infu_pAdeA_SpeI_R	AATCCATATGACTAGTGATACATGAGCTTCGGTG
Infu-pBARI-HindIII-F	TGACCATGATTACGCCAAGCTTTCAAGAGCAGAATGTGAACG
Infu-pBARI-HindIII-R	CGACCTGCAGGCATGCAAGCTTGTAAGATACATGAGCTTCGG
Infu-pPTRI-HindIII-F	TGACCATGATTACGCCAAGCTTCGACTCCAATCTTCAAGAGC
Infu-pPTRI-HindIII-R	GCAGGCATGCAAGCTTGTAAGATACATGAGCTTCGG
Infu-linker-F1	GCTCGCGAGCGCGTTCCACTGCATCATCAGTCTAG
Infu-linker-R1	AACGCGCTCGCGAGCAAGTACCATACAGTACCGCG
Infu-linker-F2	TCGCGTGCGCGTTTACCCATCATGGTGTTTTGATC
Infu-linker-R2	TAAACGCGCACGCGACATTAATCCGGATCCTTTCC
Infu-linker-pUSA-F	GCTCGCGAGCGCGTTCGATATCATGGTGTTTTGATC
Infu-linker-pUSA-R	AACGCGCTCGCGAGCCTTTCCTATAATAGACTAGCGTG
iNOS_F	GGCAGCCTGTGAGACCTTTG
iNOS_R	GCATTGGAAGTGAAGCGTTTC
TNF-αF	TTCTGTCTACTGAACTTCGGGGTGATCGGTCC
TNF-αR	GTATGAGATAGCAAATCGGCTGACGGTGTGGG
IL-6F	TCCAGTTGCCTTCTTGGGA
IL-6R	GTGTAATTAAGCCTCCGACTTG
COX-2F	TGAGTACCGCAAACGCTTCTC

COX-2R	TGGACGAGGTTTTTCCACCAG
IL-1βF	GAAAGACGGCACACCCACCCT
IL-1βR	GCTCTGCTTGTGAGGTGCTGATGTA
GAPDH-F	CATGACCACAGTCCATGCCATCAC
GAPDH-R	TGAGGTCCACCACCCTGTTGCTGT

Table S2. Plasmids constructed in this study

Plasmid	Inserts	Primer 1	Primer 2	PCR Template	Vector
pUSA-*ampA*	*ampA*	pUSA_*A*-F	pUSA_*A*-R	gDNA	pUSA digested with KpnI and SmaI
pTAex3-*ampB*	*ampB*	pTAex3_*B*-F	pTAex3_*B*-R	gDNA	pTAex3 digested with KpnI and SmaI
pUSA-*ampC*	*ampC*	pUSA_*C*-F	pUSA_*C*-R	gDNA	pUSA digested with KpnI and SmaI
pUSA-*ampD*	*ampD*	pUSA_*D*-F	pUSA_*D*-R	gDNA	
pTAex3-*ampE*	*ampE*	pTAex3_*E*-F	pTAex3_*E*-R	gDNA	pTAex3 digested with KpnI and SmaI
pTAex3-*ampF*	*ampF*	pTAex3_*F*-F	pTAex3_*F*-R	gDNA	
pTAex3-*ampG*	*ampG*	pTAex3_*G*-F	pTAex3_*G*-R	gDNA	
pTAex3-*ampH*	*ampH*	pTAex3_*H*-F	pTAex3_*H*-R	gDNA	
pTAex3-*ampI*	*ampI*	pTAex3_*I*-F	pTAex3_*I*-R	gDNA	
pTAex3-*ampJ*	*ampJ*	pTAex3_*J*-F	pTAex3_*J*-R	gDNA	
pUSA-*ampA+C*	*ampA*-T*amyB*	pUSA_*A*-F	Infu-linker- pUSA-R	pUSA-*ampA*	pUSA digested with KpnI and SmaI
	P*amyB*-*ampC*	Infu-linker- pUSA-F	pUSA_*C*-R	pUSA-*ampC*	
pAdeA-*ampE+F*	P*amyB*-*ampE*-T*amyB*	Infu_pAdeA_SpeI_F	Infu-linker-R1	pTAex3-*ampE*	pAdeA digested with SpeI
	P*amyB*-*ampF*-T*amyB*	Infu-linker-F1	Infu_pAdeA_SpeI_R	pTAex3-*ampF*	
pPTRI-*ampD+G*	P*amyB*-*ampD*-T*amyB*	Infu-pPTRI-HindIII-F	Infu-linker-R1	pUSA-*ampD*	pPTRI digested with HindIII
	P*amyB*-*ampG*-T*amyB*	Infu-linker-F1	Infu-pPTRI-HindIII-R	pTAex3-*ampG*	
pBARI-*ampH+I+J*	P*amyB*-*ampH*-T*amyB*	Infu-pBARI-HindIII-F	Infu-linker-R1	pTAex3-*ampH*	pBARI digested with HindIII
	P*amyB*-*ampI*-T*amyB*	Infu-linker-F1	Infu-linker-R2	pTAex3-*ampI*	
	P*amyB*-*ampJ*-T*amyB*	Infu-linker-F2	Infu-pBARI-HindIII-R	pTAex3-*ampJ*	

Table S3. *A. oryzae* transformants constructed in this study

Strains	Host strain	Plasmids used for transformation
AmpB	*A. oryzae* NSAR1	pTAex3-*ampB*
AmpBC	*A. oryzae* NSAR1	pTAex3-*ampB*, pUSA-*ampC*
AmpABCDEFG	*AmpABC*	pAdeA-*ampE+F*, pPTRI-*ampD+G*
AmpABCDEFGHIJ	*AmpABCDEFG*	pBARI-*ampH+I+J*

Table S4. ^{1}H NMR and ^{13}C NMR data of udagawanone A (**3**) (400 and 100 MHz) (inCD_3OD-d_4, δ in ppm, *J* in Hz)

No	δ_H,(*J* in Hz)	$\delta_{C,}$ type
2		166.9, C
3		111.5, C
4		172.2, C
5		110.5, C
6		155.6, C
7	6.45, dd (15.3, 1.4)	121.4, CH
8	6.67, dq (15.4, 6.9)	135.9, CH
9	1.95, dd (6.9, 1.2)	18.7, CH_3
10	4.53, s	55.2, CH_2
11	1.99, s	9.6, CH_3
12	4.08, s	62.4, CH_3

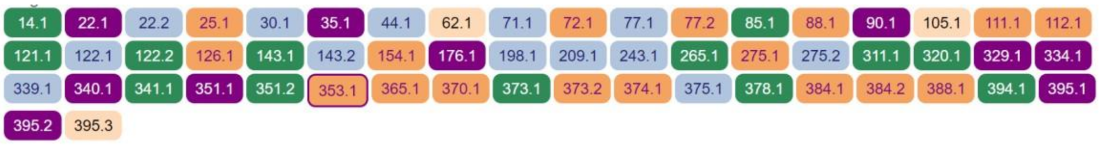

Figure S1. The antiSMASH (fungal version) result of *Amphichorda felina* SYSU-MS7908

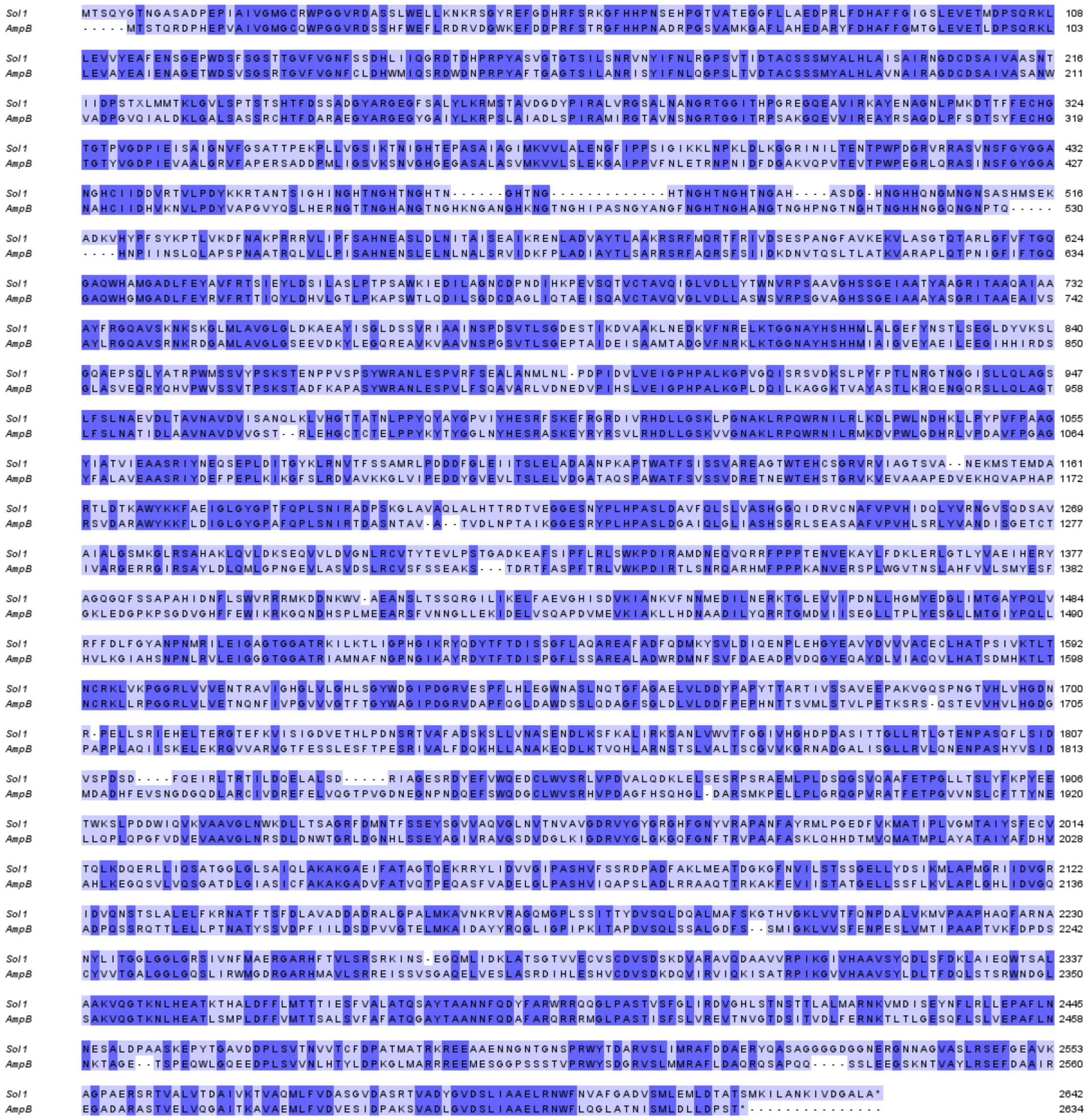

Figure S2. Amino acid sequence alignment of AmpB with Sol1. The sequences are aligned using the program Clustal Omega and Jalview.

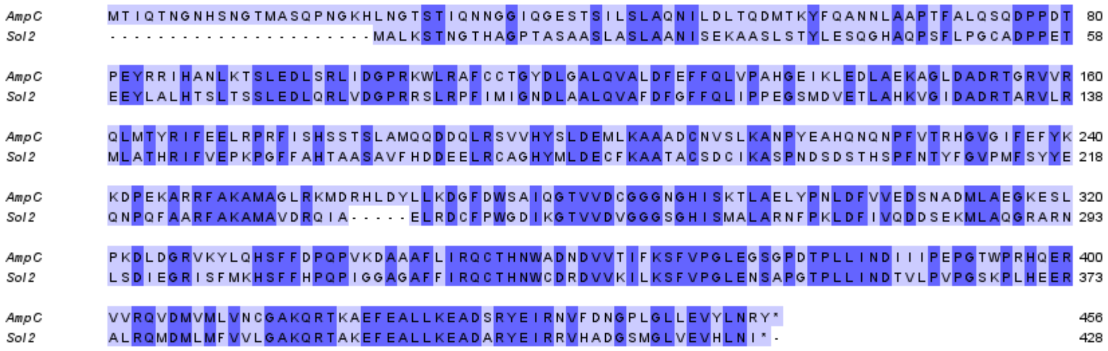

Figure S3. Amino acid sequence alignment of AmpC with Sol2. The sequences are aligned using the program Clustal Omega and Jalview.

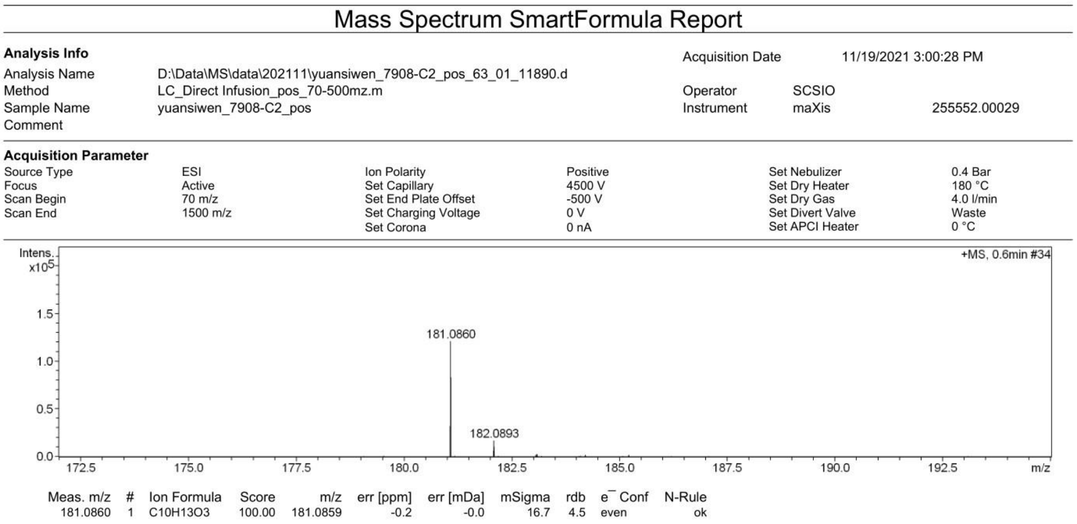

Figure S4. HRESIMS spectrum for amphichopyrone A (**1**)

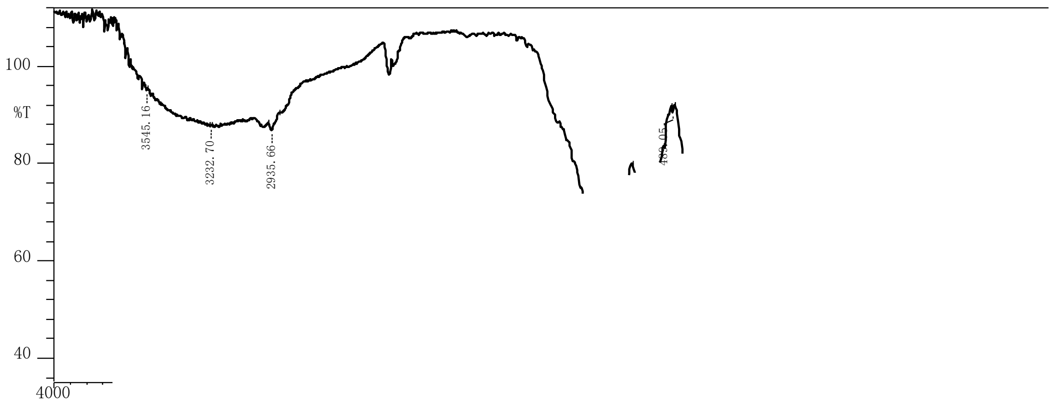

Figure S5. The IR spectrum of Amphichopyrone A (**1**)

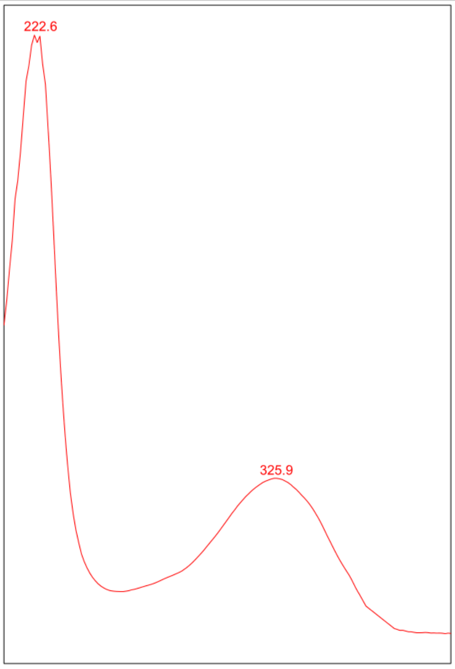

Figure S6. The UV spectrum of Amphichopyrone A (**1**)

Mass Spectrum SmartFormula Report

Analysis Info

Analysis Name	D:\Data\MS\data\202111\yuansiwen_7908-C1_pos_62_01_11889.d	Acquisition Date	11/19/2021 2:57:01 PM
Method	LC_Direct Infusion_pos_70-500mz.m	Operator	SCSIO
Sample Name	yuansiwen_7908-C1_pos	Instrument	maXis 255552.00029
Comment			

Acquisition Parameter

Source Type	ESI	Ion Polarity	Positive	Set Nebulizer	0.4 Bar
Focus	Active	Set Capillary	4500 V	Set Dry Heater	180 °C
Scan Begin	70 m/z	Set End Plate Offset	-500 V	Set Dry Gas	4.0 l/min
Scan End	1500 m/z	Set Charging Voltage	0 V	Set Divert Valve	Waste
		Set Corona	0 nA	Set APCI Heater	0 °C

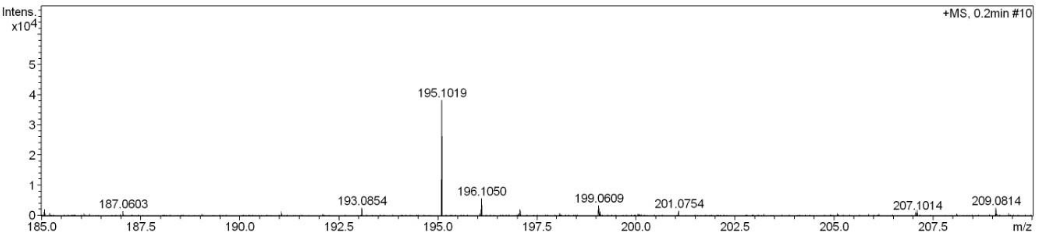

Meas. m/z	#	Ion Formula	Score	m/z	err [ppm]	err [mDa]	mSigma	rdb	e⁻ Conf	N-Rule
195.1019	1	C11H15O3	100.00	195.1016	1.5	0.3	16.2	4.5	even	ok

Figure S7. HRESIMS spectrum for amphichopyrone B (**2**)

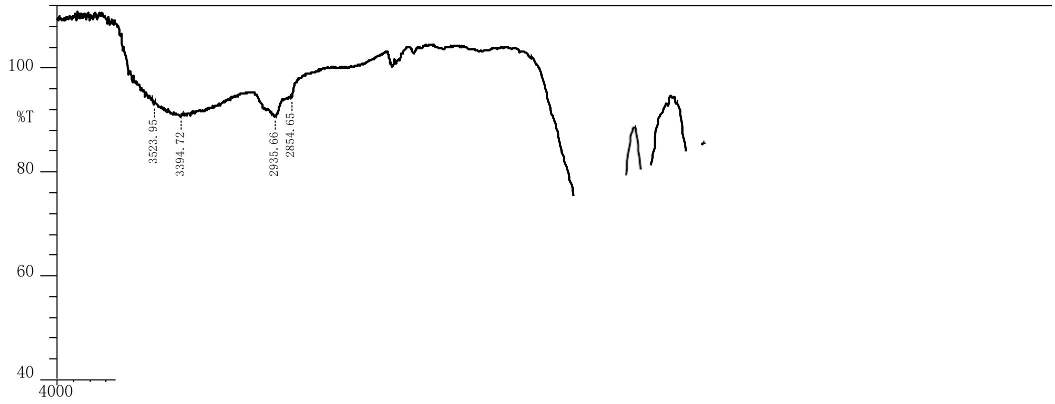

Figure S8. The IR spectrum of Amphichopyrone B (**2**)

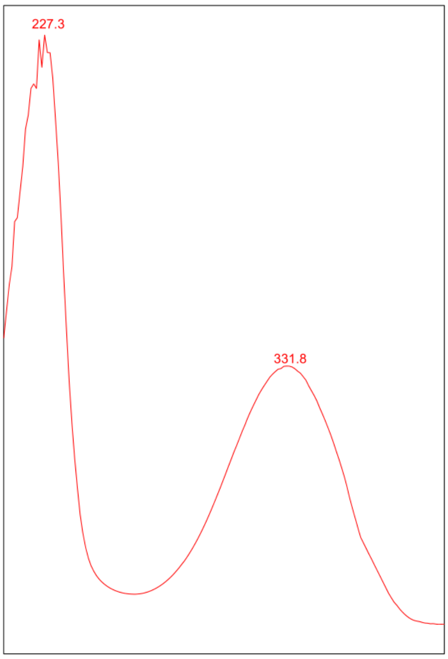

Figure S9. The UV spectrum of Amphichopyrone B (**2**)

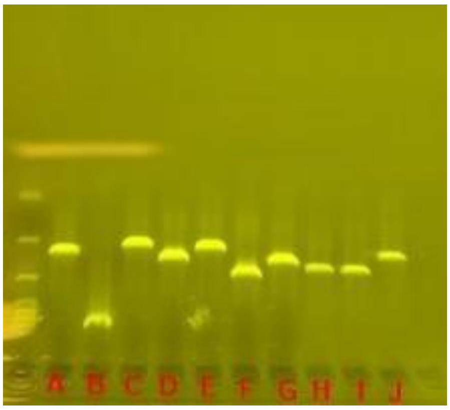

Figure S10. PCR analysis for the AO-*ampABCDEFGHIJ* construct.

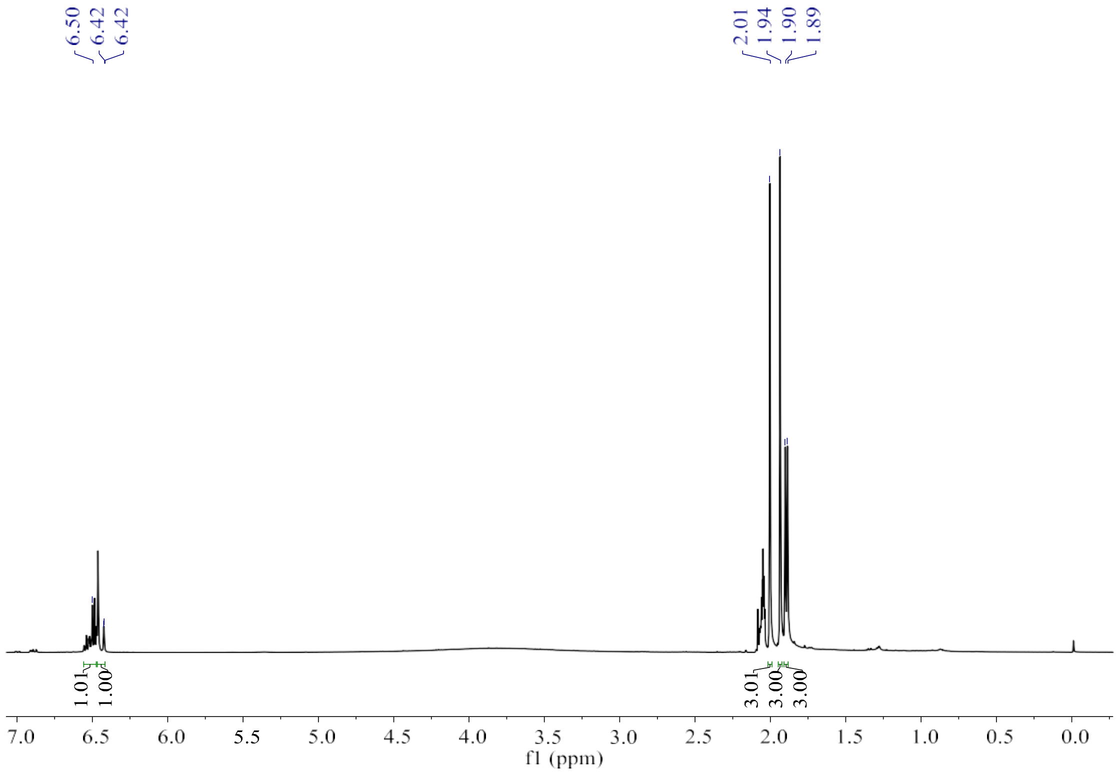
6.50
6.42
6.42
2.01
1.94
1.90
1.89
1.01
1.00
3.01
3.00
3.00
7.0 6.5 6.0 5.5 5.0 4.5 4.0 3.5 3.0 2.5 2.0 1.5 1.0 0.5 0.0
f1 (ppm)

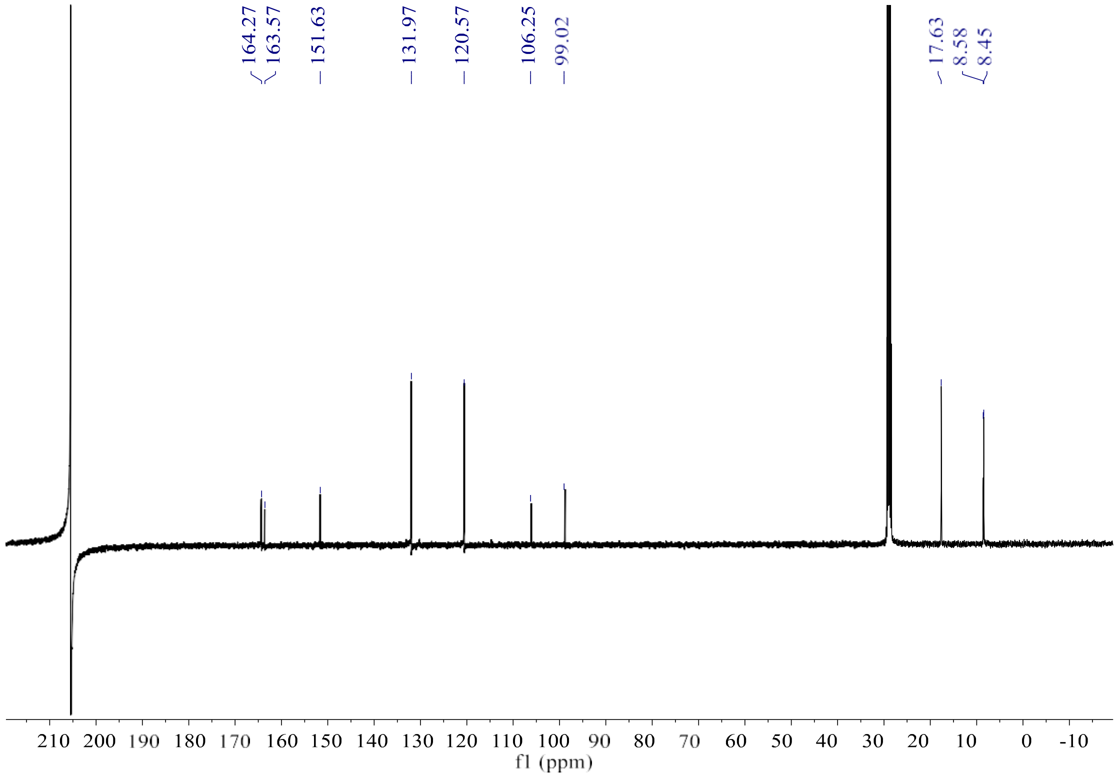
164.27
163.57
151.63
131.97
120.57
106.25
99.02
17.63
8.58
8.45
210 200 190 180 170 160 150 140 130 120 110 100 90 80 70 60 50 40 30 20 10 0 -10
f1 (ppm)

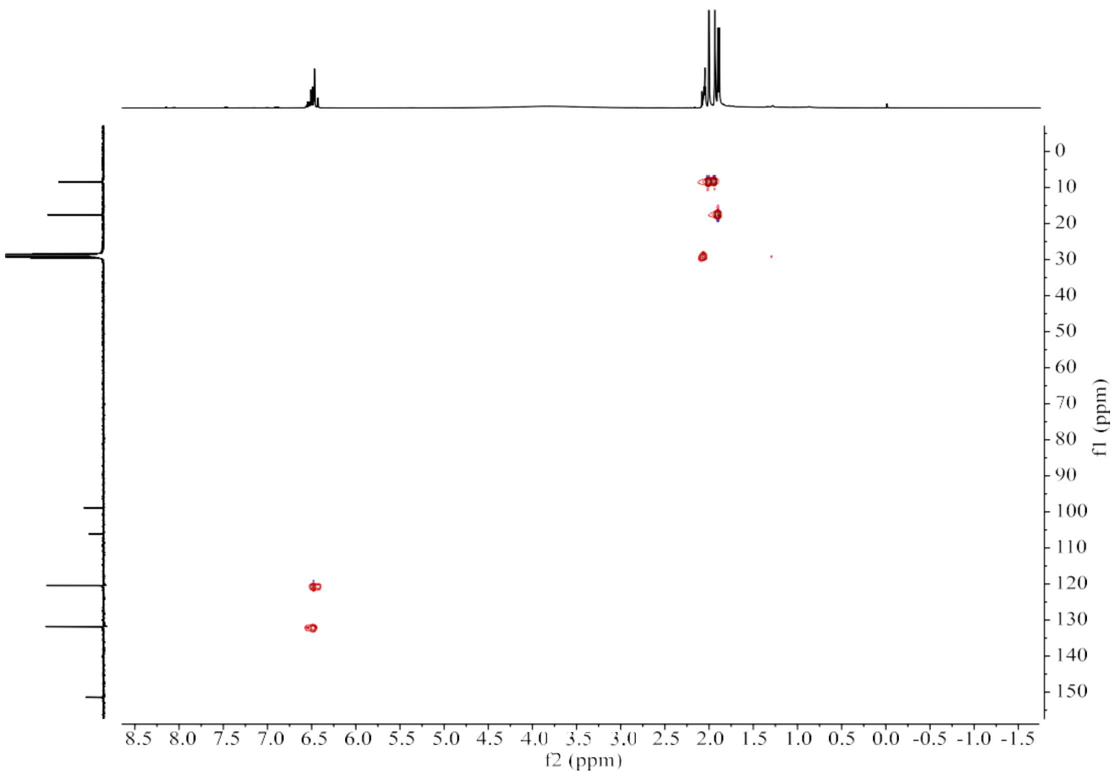

8.5 8.0 7.5 7.0 6.5 6.0 5.5 5.0 4.5 4.0 3.5 3.0 2.5 2.0 1.5 1.0 0.5 0.0 -0.5 -1.0 -1.5
f2 (ppm)
0 10 20 30 40 50 60 70 80 90 100 110 120 130 140 150
f1 (ppm)

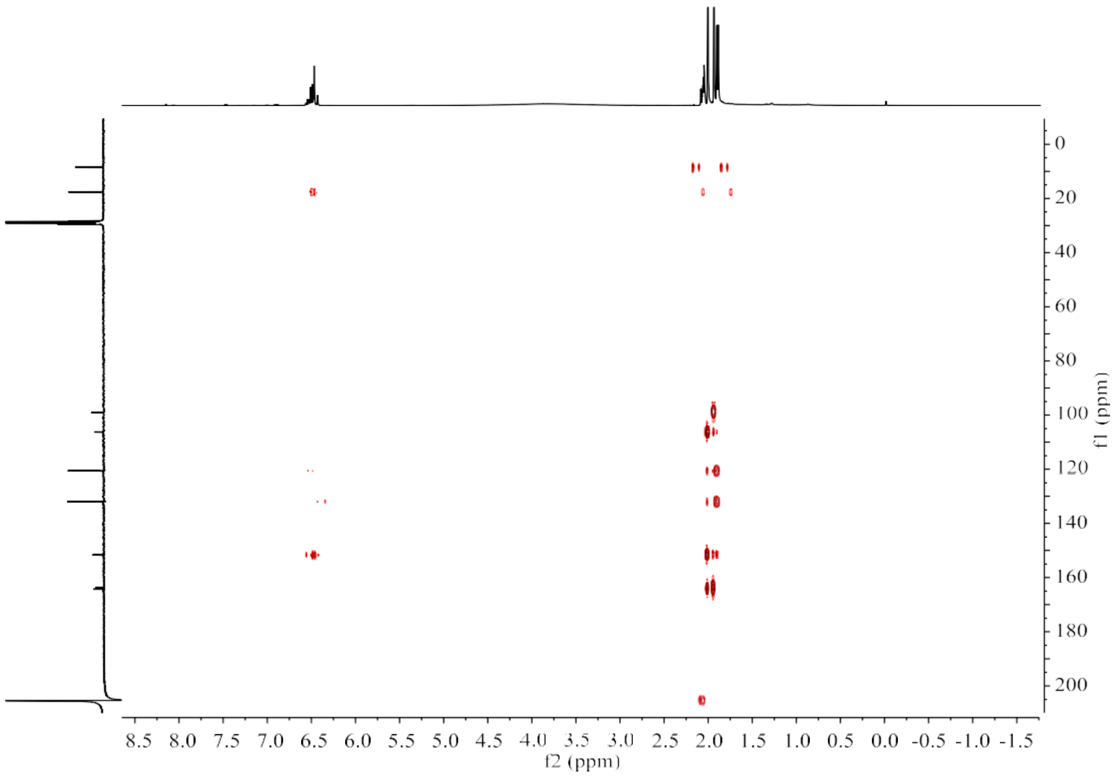

8.5 8.0 7.5 7.0 6.5 6.0 5.5 5.0 4.5 4.0 3.5 3.0 2.5 2.0 1.5 1.0 0.5 0.0 -0.5 -1.0 -1.5
f2 (ppm)
0 20 40 60 80 100 120 140 160 180 200
f1 (ppm)

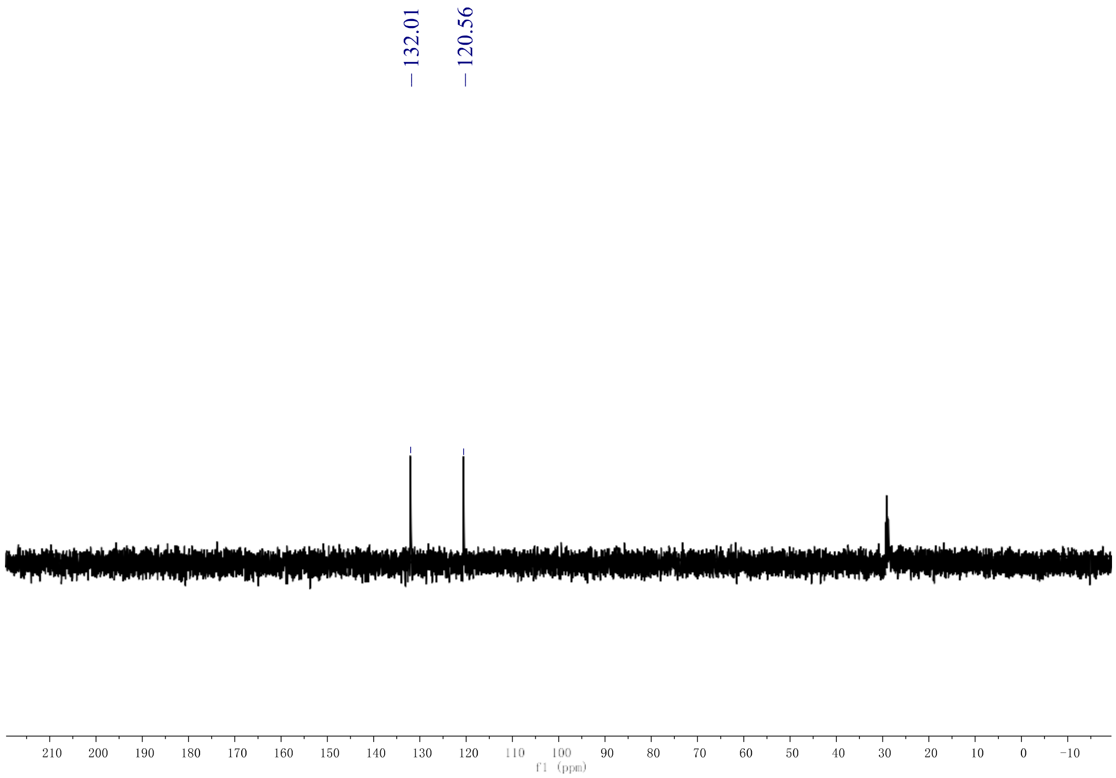

DEPT90 spectrum for amphichopyrone A (**1**) (400 MHz, acetone-d_6)

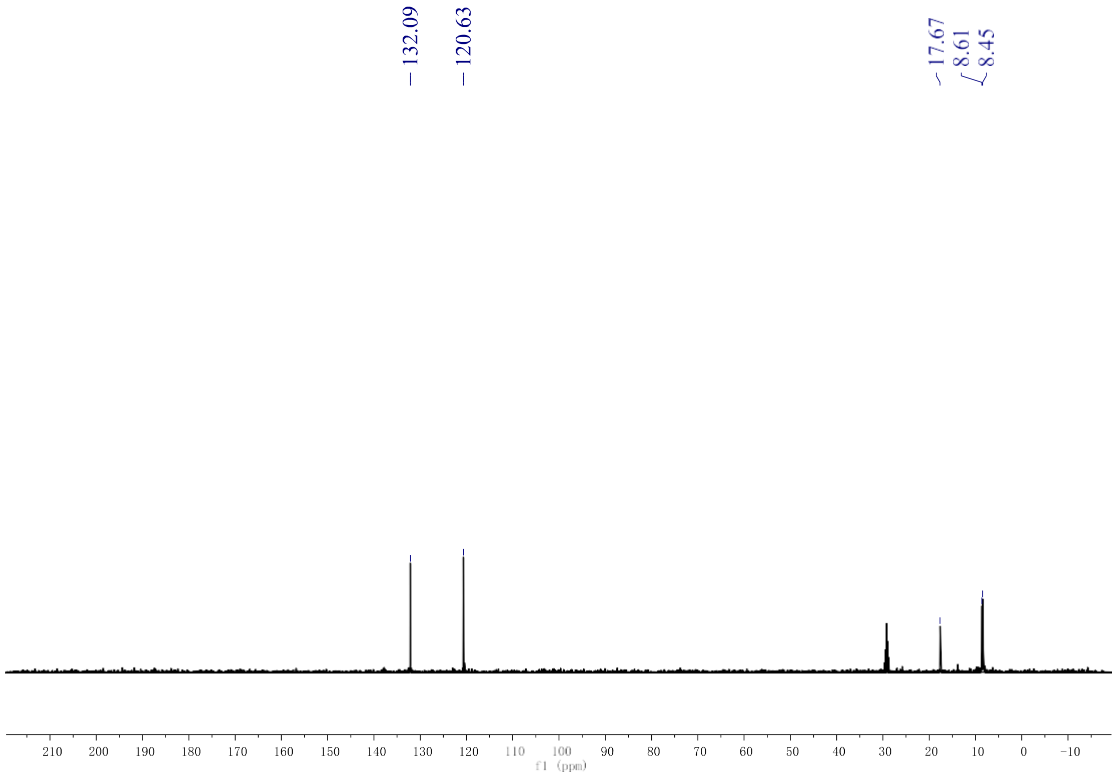

DEPT135 spectrum for amphichopyrone A (**1**) (400 MHz, acetone-d_6)

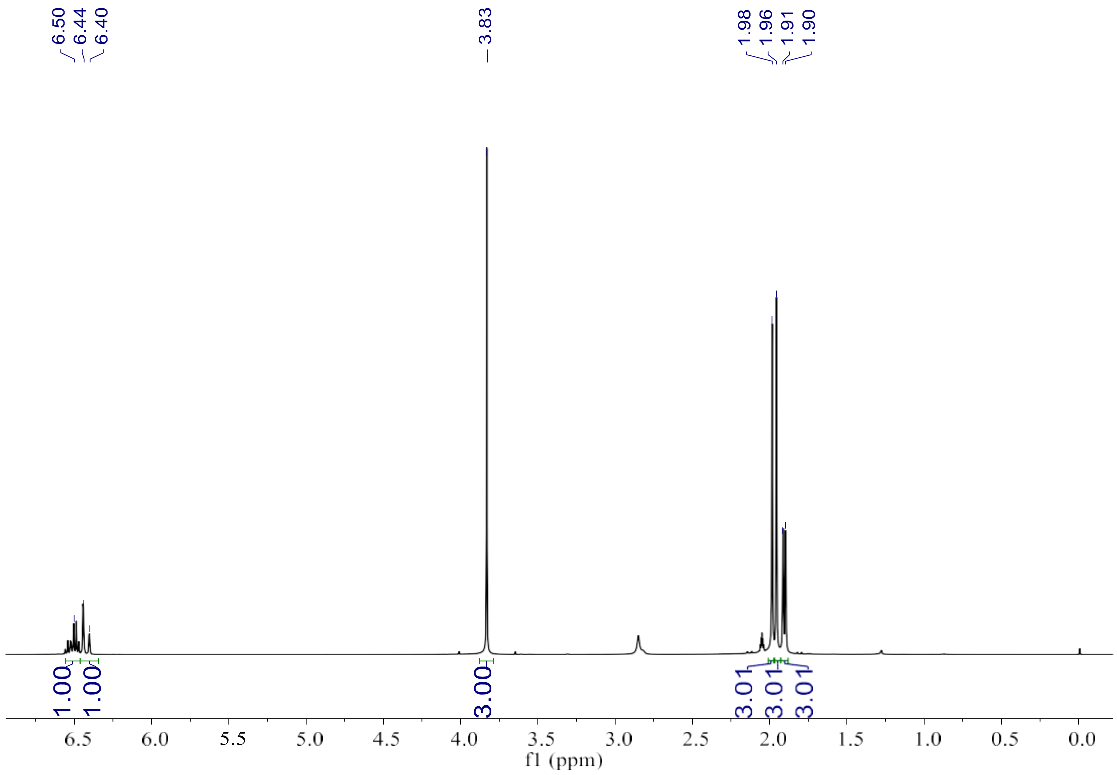
6.50
6.44
6.40
3.83
1.98
1.96
1.91
1.90
1.00
1.00
3.00
3.01
3.01
3.01
6.5 6.0 5.5 5.0 4.5 4.0 3.5 3.0 2.5 2.0 1.5 1.0 0.5 0.0
f1 (ppm)

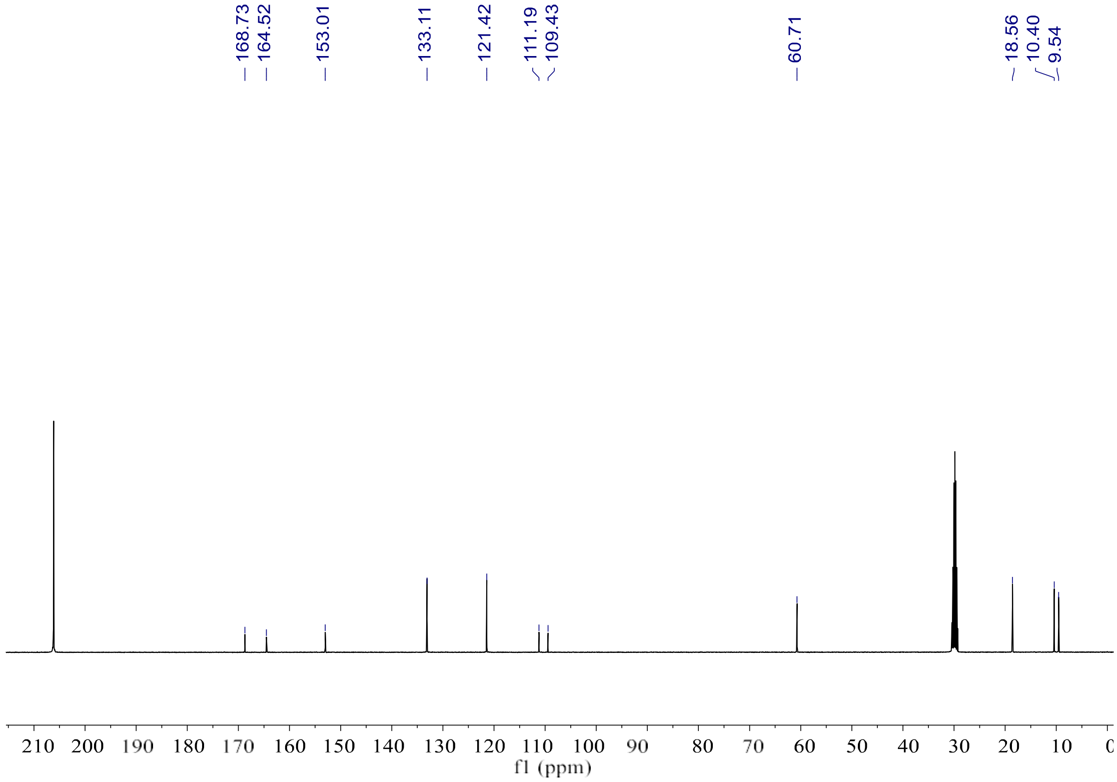
168.73
164.52
153.01
133.11
121.42
111.19
109.43
60.71
18.56
10.40
9.54
210 200 190 180 170 160 150 140 130 120 110 100 90 80 70 60 50 40 30 20 10
f1 (ppm)

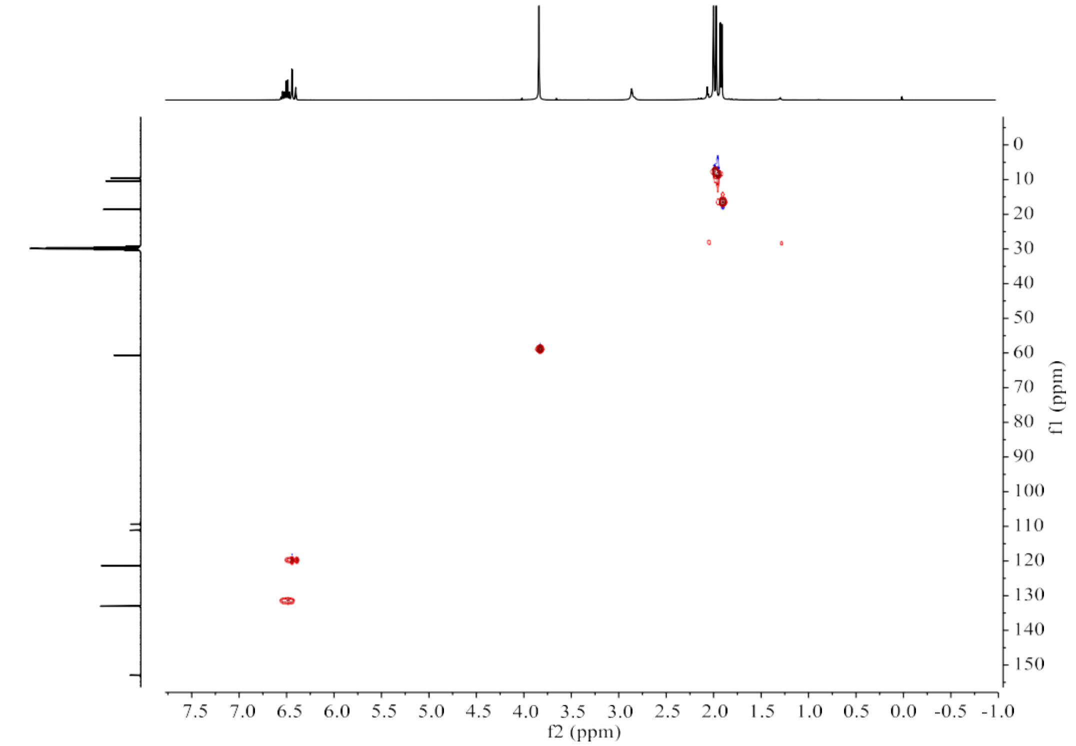

f2 (ppm)
f1 (ppm)

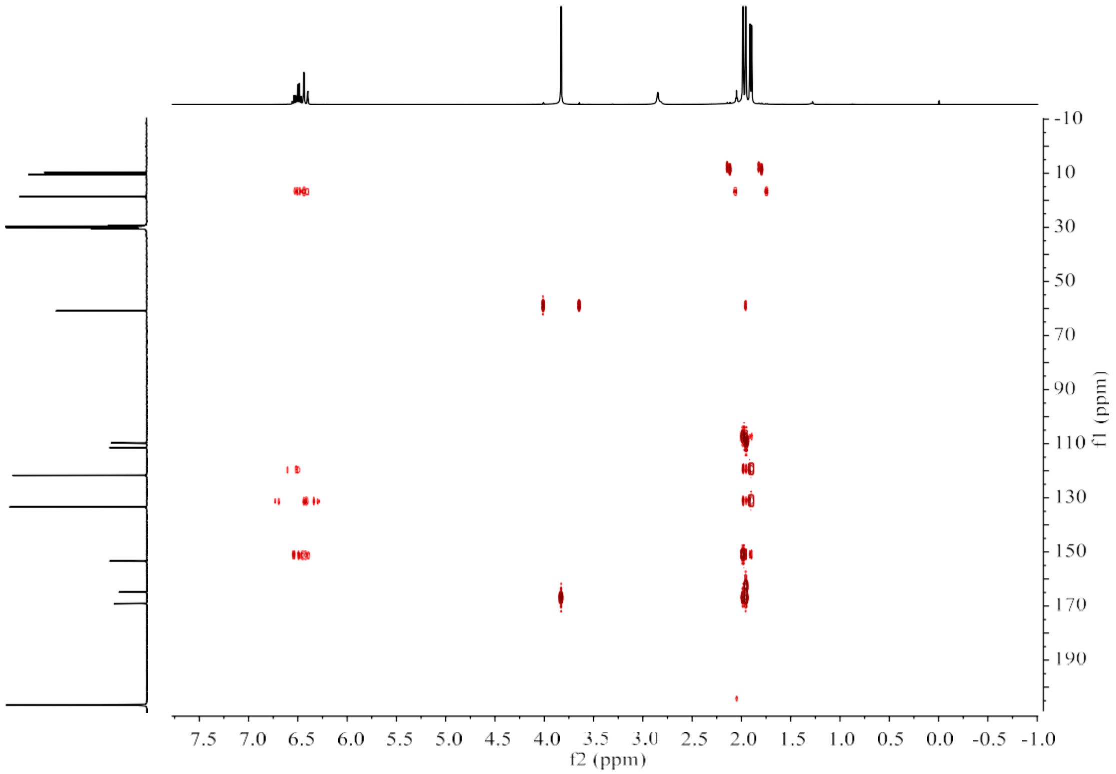

f2 (ppm)
f1 (ppm)

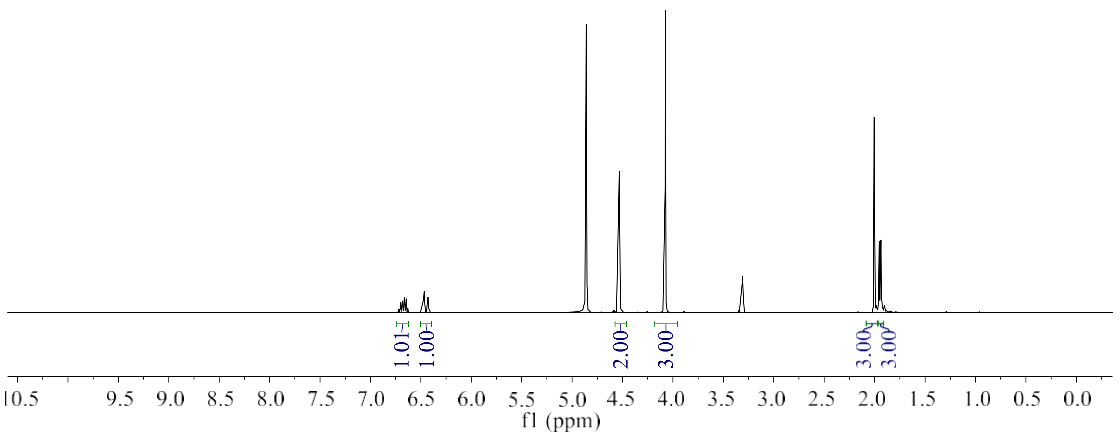
1.01
1.00
2.00
3.00
3.00
3.00
10.5 9.5 9.0 8.5 8.0 7.5 7.0 6.5 6.0 5.5 5.0 4.5 4.0 3.5 3.0 2.5 2.0 1.5 1.0 0.5 0.0
f1 (ppm)

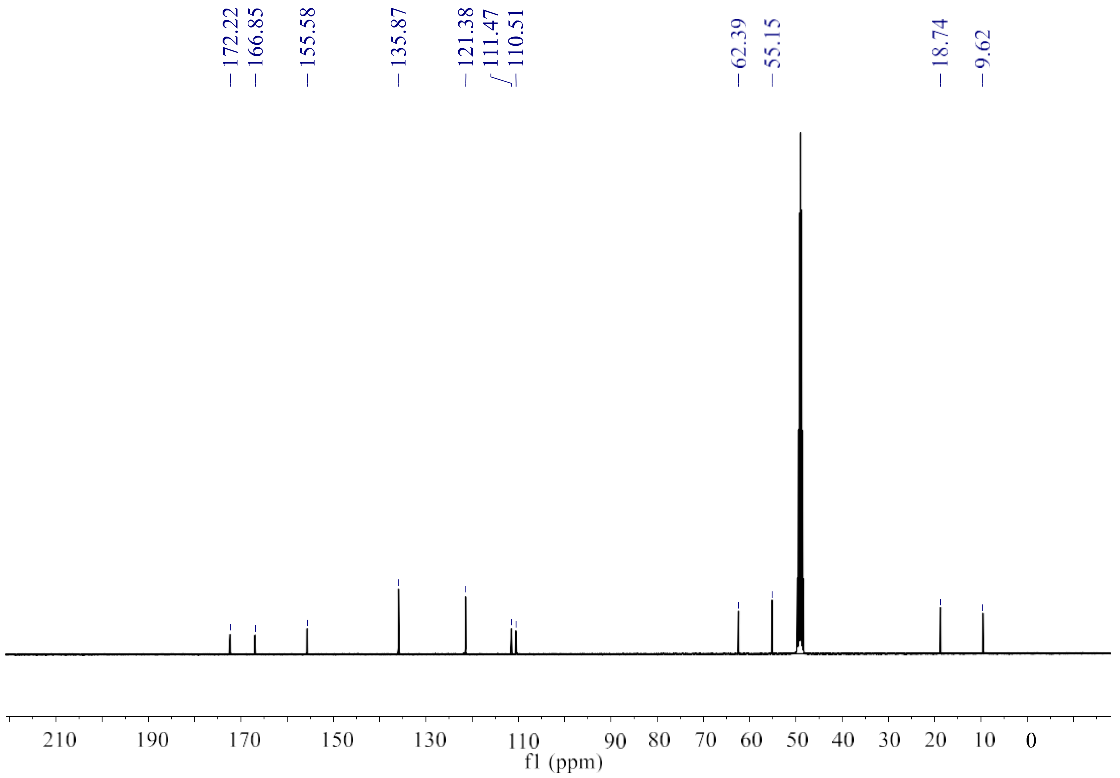
172.22
166.85
155.58
135.87
121.38
111.47
110.51
62.39
55.15
18.74
9.62
210 190 170 150 130 110 90 80 70 60 50 40 30 20 10 0
f1 (ppm)

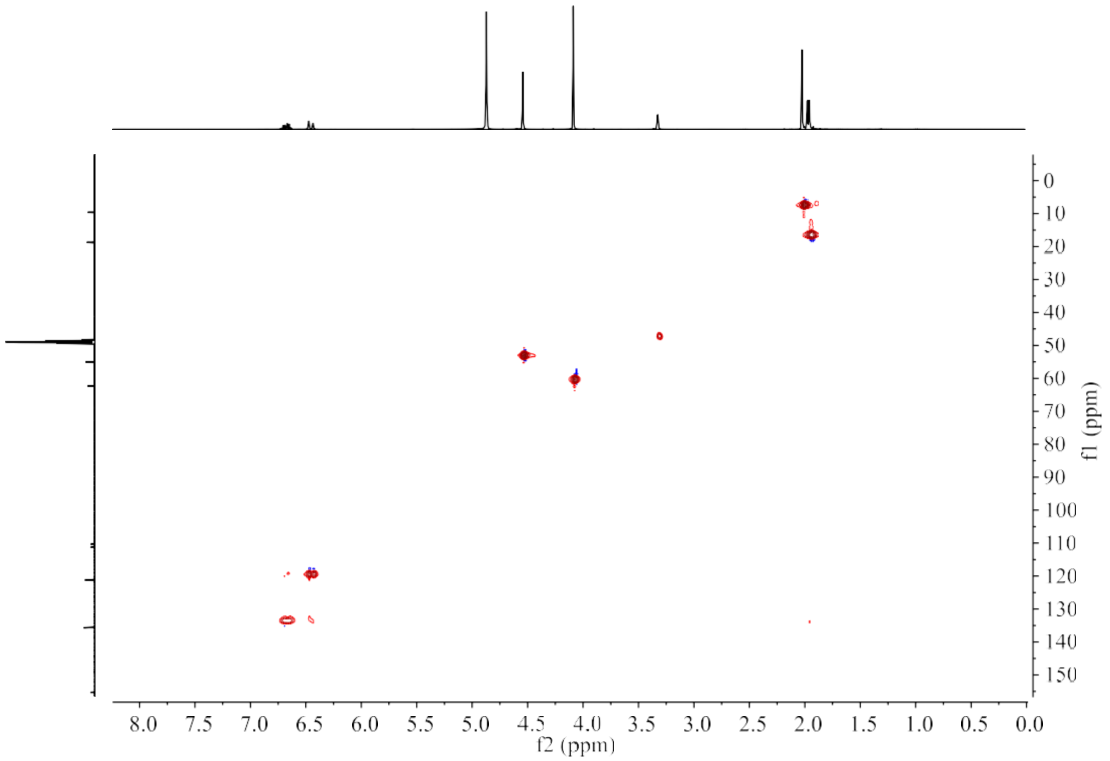
0
10
20
30
40
50
60
70
80
90
100
110
120
130
140
150
f1 (ppm)
8.0 7.5 7.0 6.5 6.0 5.5 5.0 4.5 4.0 3.5 3.0 2.5 2.0 1.5 1.0 0.5 0.0
f2 (ppm)

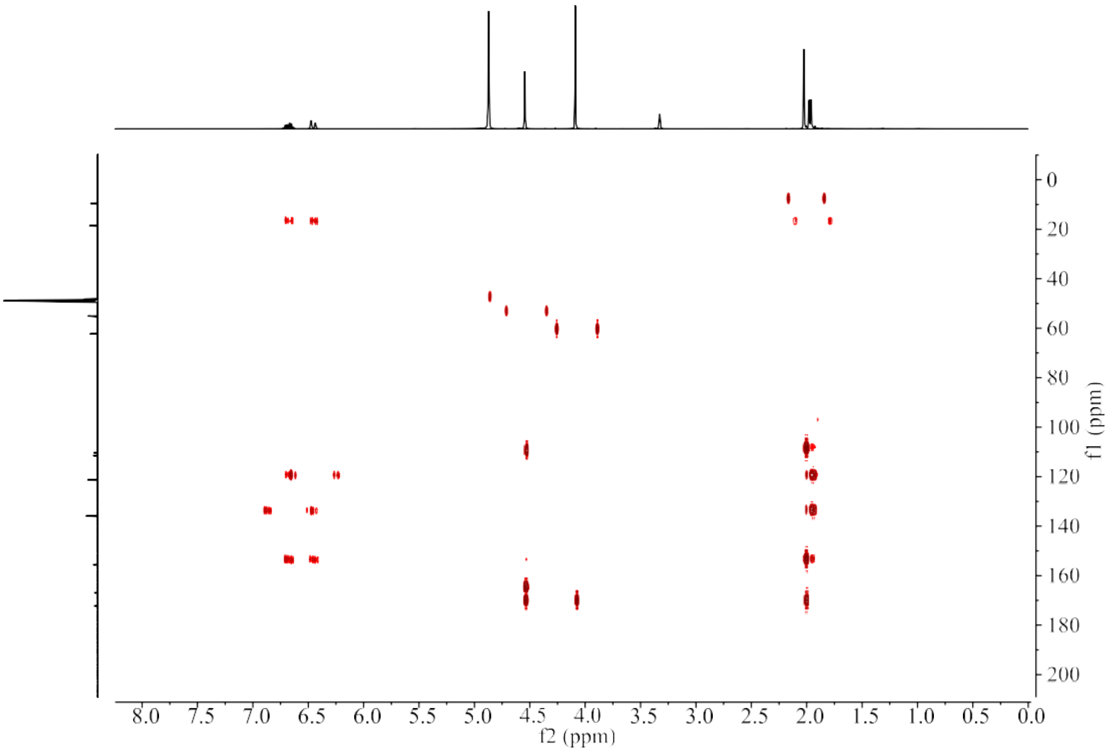
0
20
40
60
80
100
120
140
160
180
200
f1 (ppm)
8.0 7.5 7.0 6.5 6.0 5.5 5.0 4.5 4.0 3.5 3.0 2.5 2.0 1.5 1.0 0.5 0.0
f2 (ppm)
